# Electrolyzed saline as an alternative to chlorhexidine: Antimicrobial and biofilm volume outcomes in a 4‐day non‐brushing randomized controlled clinical trial

**DOI:** 10.1002/jper.70070

**Published:** 2026-02-02

**Authors:** Katja Povšič, Haris Munjaković, Naiera Zayed, Wim Teughels, Katja Seme, Aleš Fidler, Rok Gašperšič

**Affiliations:** ^1^ Department of Oral Medicine and Periodontology Faculty of Medicine University of Ljubljana Ljubljana Slovenia; ^2^ Department of Oral Medicine and Periodontology University Medical Centre Ljubljana Ljubljana Slovenia; ^3^ Department of Oral Health Sciences University of Leuven (KU Leuven) Leuven Belgium; ^4^ Department of Microbiology and Immunology Faculty of Pharmacy Menoufia University Menoufia Egypt; ^5^ Institute of Microbiology and Immunology Faculty of Medicine University of Ljubljana Ljubljana Slovenia; ^6^ Department of Endodontics and Operative Dentistry Faculty of Medicine University of Ljubljana Ljubljana Slovenia; ^7^ Department of Restorative Dentistry and Endodontics University Medical Centre Ljubljana Ljubljana Slovenia

**Keywords:** antiseptics, chlorhexidine, dental biofilm, electrolyzed saline, mouthwash, oral microbiology

## Abstract

**Background:**

The biofilm‐inhibitory effects of electrolyzed saline (EOS) vary widely due to differences in formulations and treatment methods. This study analyzed the impact of EOS on key oral microbial species and used serial 3D‐intraoral scans to evaluate its effects on *de novo* biofilm formation.

**Methods:**

This was a double‐blind, randomized, placebo‐controlled, cross‐over, 4‐day, non‐brushing, plaque‐regrowth study on periodontally healthy individuals. Each subject participated in three trial arms. During each 4‐day arm, the subjects refrained from mechanical oral hygiene. Instead, they rinsed their oral cavities twice‐daily with EOS, 0.12% chlorhexidine (CHX), or placebo. *De novo* plaque accumulation after 4 days was assessed as the primary outcome using the Turesky Modification of the Quigley‐Hein Plaque Index (TMQHPI) and the volumetric plaque index (VPI). qPCR analyses of key‐microbial species and measurements of active‐matrix‐metalloproteinase‐8 (aMMP‐8) in gingival crevicular fluid were performed to complement the clinical data.

**Results:**

The biofilm‐inhibitory effect of CHX was superior to that of EOS at the level of TMQHPlI and VPI, although both significantly reduced biofilms compared with placebo. The broad‐spectrum antimicrobial effect of CHX caused significant reductions in overall bacterial loads, while the action of EOS was more selective. Both CHX and EOS markedly reduced the bacterial loads of *Tannerella forsythia*; CHX remained more effective against *Treponema denticola*. In contrast, only EOS demonstrated stronger antimicrobial effects against *Fusobacterium nucleatum* and *Prevotella intermedia* while showing no significant impact on periodontal commensals. No significant effects on aMMP‐8 were observed.

**Conclusions:**

EOS showed substantial, but inferior biofilm‐inhibitory, effects compared with CHX. However, EOS had more selective and dysbiosis‐controlling effects than CHX.

The clinical trial was registered at Clintrials.gov under no. NCT05709015.

**Plain language summary:**

This study investigated the effectiveness of electrolyzed saline (EOS) in preventing biofilm build‐up on teeth. The efficacy of EOS mouthwash was compared with a positive control (chlorhexidine, CHX) and a negative control (distilled water). A method based on 3D scans of teeth was used to measure changes in the biofilm volume. In the study, participants stopped brushing their teeth for 4 days and rinsed their mouths twice daily with either EOS, CHX, or a placebo instead. They were then assessed for biofilm levels and changes in oral bacteria numbers. The results showed that CHX was more effective than EOS in reducing biofilm, although both were more effective than placebo. Chlorhexidine significantly lowered harmful bacteria but also negatively affected the beneficial bacteria. On the other hand, EOS also reduced specific harmful bacteria, but did not lower the beneficial ones as much. Overall, while EOS was less effective at reducing biofilm than CHX, it was better at maintaining a healthy balance of oral bacteria.

## 1 INTRODUCTION

The primary etiologic factors of gingivitis and periodontitis are microbial biofilms.^1^ Treatment predominately includes mechanical measures aimed at reducing oral biofilms, often with the adjunct use of antimicrobial mouthwashes.^2^, ^3^, ^4^ Despite such measures, microbial communities recolonize the oral cavity within a very short time, thus impacting the host–immune response, which can be unfavorable (pro‐inflammatory) or favorable (health‐promoting).^5^, ^6^ Since most oral antimicrobial agents are non‐selective and target both symbiosis and dysbiosis‐associated species, their elimination and recolonization should be closely monitored.

Chlorhexidine digluconate (CHX) remains the gold standard among biofilm‐inhibitory agents.^7^ Since CHX targets both oral commensals and pathobionts non‐selectively,^8^ alternative anti‐biofilm agents with selective antimicrobial characteristics are being developed. Electrolyzed saline (EOS), a chlorine‐based antimicrobial agent containing hypochlorous acid (HOCl), has recently demonstrated its efficacy by inhibiting the recolonization of periodontal pathobionts and, more importantly, ensuring the dominance of *Streptococci* as commensal species in in vitro subgingival multispecies biofilms.^8^

Biofilm deposits have traditionally been evaluated visually using plaque‐disclosing agents (PDAs),^9^ most frequently with the Turesky Modification of the Quigley‐Hein Plaque Index (TMQHPlI).^10^, ^11^ However, conventional clinical methods have several limitations, including low sensitivity, variability, subjectiveness, and imprecision. To overcome them, digital approaches based on intraoral scanning (IOS) have been proposed.^12^ The superimposition of serial 3D‐dental models enables the quantification of biofilms, as well as their visualization using color‐coded maps without PDAs.

The aim of this study was to evaluate the biofilm‐inhibitory efficacy of an EOS mouthwash in a 4‐day non‐brushing model, compared with CHX. We hypothesized that EOS would be less effective than CHX in preventing biofilm formation as evaluated by a digital volumetric approach but would alter the microbial composition of biofilms more favorably by inhibiting the recolonization of pathobionts and preserving commensal species.

## 2 MATERIALS AND METHODS

The study was performed from January–February 2023 at the Department of Oral Medicine and Periodontology, University Dental Clinic of Ljubljana, Slovenia. It was approved by the National Medical Ethic Committee of the Republic of Slovenia (0120‐444/2022/3) and registered at Clintrials.gov (NCT05709015). Written and oral explanations regarding the research protocol were given to eligible volunteers. All subjects filled out written informed consent forms prior to participation. The study was performed in accordance with the principles of the Declaration of Helsinki.

### 2.1 Experimental design

The study was designed as a double‐blind, 3‐arm cross‐over, randomized, single‐center, 4‐day non‐brushing plaque‐regrowth model.^13^ During a test interval of 4 consecutive days, the subjects were instructed to cease all mechanical oral hygiene practices and to use a mouthwash instead. To avoid any carryover effects, the first test arm was followed by a minimum of 7 days. The second 4‐day non‐brushing test interval was subsequently begun with a new mouthwash, followed by the second washout period, and this was followed by the third 4‐day non‐brushing test interval. During each washout, the subjects were instructed to resume their normal mechanical oral hygiene routine (brushing with toothpaste and flossing with no standardized products), to refrain from using other oral chemical formulations, mouthwashes or antibacterial oral products, and to maintain their usual diet.

Each subject was asked to complete all three of its arms, thus testing all three of the examined formulations (Figure S1 in the online *Journal of Periodontology*): EOS with 200 ppm of free chlorine (pH 7.0), 0.12% CHX (positive control), and placebo (distilled water; negative control).

### 2.2 Study population

Consecutively selected, systemically healthy (no known systemic illness, no medication), non‐smoking volunteers with no periodontal pathologies^14^ were included according to the inclusion/exclusion criteria (Table S1 in the online *Journal of Periodontology*).

### 2.3 Inclusion assessment and baseline examination

At baseline, all volunteers were assessed for potential inclusion. After filling out a health‐assessment questionnaire, a full periodontal examination was performed by a calibrated^15^ examiner (K. P.) using a manual Williams probe (POW6, Hu‐Friedy, Chicago, IL, USA). Periodontal parameters were evaluated at six sites per tooth: TMQHPlI, Lobene's modified gingival index (MGI),^16^ probing pocket depth (PD), gingival recession (REC), and bleeding on probing (BOP). Clinical attachment loss (CAL) was calculated as the sum of PD and REC.

### 2.4 Clinical protocol

After the initial examination, qualifying subjects were randomly assigned to their individual sequence of mouthwash use during each of the three study arms. The CONSORT flow diagram is presented in Figure S2.

All subjects received professional supragingival dental prophylaxis and tooth polishing using piezoelectric ultrasonic instruments (PiezoLED ultrasonic scaler, Piezo Scaler tip 203; KaVo Dental), and prophylactic bristle brushes until all biofilm/stain/calculus were removed. Interdental spaces were cleaned using floss (Essential Floss and Super Floss; Oral‐B). Next, maxillary and mandibular digital models were obtained using intraoral optical scanning (Trios 4; 3shape), to serve as baseline digital scans. The subgingival biofilm was sampled for microbiological analysis. Gingival crevicular fluid (GCF) was sampled for the measurement of active matrix metalloproteinase‐8 (aMMP‐8). Subjects were then instructed to cease all mechanical oral hygiene practices for 4 consecutive days and to use their assigned mouthwash twice daily (after breakfast and dinner) instead: upon each use, they were asked to first rinse their oral cavities with tap water for 1 min, followed immediately by 1 min of rinsing with 15 mL of the assigned mouthwash. The mouthwashes were to be kept in a refrigerator.

After 4 days (T4), that is, at the end of each study arm, the subjects were recalled for a follow‐up. Adverse effects and organoleptic characteristics of the mouthwashes were recorded (Figure S3). Packaging was assessed for compliance control. Each subject's oral cavity was examined for mucous membrane changes; gingival inflammation was assessed using the MGI. After application of a two‐color system disclosing agent (PlaqueFinder 260 disclosing solution; Curaprox) using cotton pellets, participants rinsed their oral cavities with 20 mL of tap water for 15 s. Maxillary and mandibular digital models were then obtained using intraoral optical scanning (Trios 4, 3shape). The digital volumetric biofilm analysis based on superimposed serial scans^15^ is described in Figure S4; the volumetric plaque index (VPI; i.e., plaque volume in mm^3^) and adjusted volumetric plaque index (AVPI; i.e., plaque volume per surface area in mm^3^/mm^2^) were used to assess biofilm volume. New biofilm formation was evaluated using the TMQHPlI at six sites per tooth, excluding third molars. TMQHPlI was assessed separately for dark (purple) colored disclosed plaque (DDP) and total (pink + purple) disclosed plaque (TDP). Next, subgingival samples were collected for microbiological and a‐MMP‐8 analysis. Finally, all subjects received professional supragingival dental prophylaxis and tooth polishing using prophylactic bristle brushes and polishing paste (Proxyt; Ivocal Vivadent). At the end of the last arm, a topical application of fluorides (Elmex Gelee 1,25% detal gel; Elmex) was performed.

### 2.5 Microbiological protocol

Subgingival biofilm samples were taken at T0 and T4 of each study arm from the buccal surfaces of four Ramfjord teeth (16, 21, 36, 41) using absorbent paper points (diameter: 0.30 mm; Maillefer) to obtain a pooled sample. After site isolation with cotton rolls and airdrying, two paper points were sequentially kept in place for 10 s. All four samples from each individual were placed into a vial containing 1 mL of reduced transport fluid. The samples were frozen at −20°C and sent to the Institute of Microbiology and Immunology, Faculty of Medicine, University of Ljubljana.

After defrosting, the samples were vigorously mixed for 30 s; each sample was centrifuged for 10 min at 6000 × *g*, and the supernatants were discarded. DNA was then extracted from pelleted bacterial cells using a QIAamp DNA Mini‐kit (Qiagen, Hilden, Germany).^17^ qPCR identification (Figure S5) of selected key oral microbial species (Table S2) was performed with a CFX96 real‐time system (Bio‐Rad, Hercules, CA, USA), using species‐specific primers and probes (Table S3).

### 2.6 aMMP‐8 analysis

Samples of GCF were obtained using Periopaper strips (Periopaper; ProFlow, Amityville, NY, USA) at the beginning and end of each study arm for a chairside aMMP‐8 analysis using a lateral‐flow test kit and ORALyser reader quantitation (PerioSafe‐Oralyzer; Dentognostics GmbH, Jena, Germany) with a limit of detection (LOD) = 20 ng/mL, according to the manufacturer's instructions (Figure S6).^18^, ^19^

### 2.7 Randomization and blinding

Randomization was performed using a computer‐generated table (randomizer.org) for a double‐blind design. The identities of the three mouthwash formulations were coded and kept by the study coordinator (H. M.); they were only revealed after the last follow‐up examination of the last subject. All mouthwashes were prepared in identical packaging and were only labelled with the subject identification number, consecutive study arm number, date of study arm commencement, and instructions.

### 2.8 Sample size calculation

Sample size calculation was performed based on the primary outcome – TMQHPlI. According to similar studies,^20^ a sample size of 15 patients was considered adequate, showing 85% of power to detect a difference of 0.50 in the TMQHPlI between the test and the negative control, considering a standard deviation of 0.66. Nevertheless, the sample size was increased by 15% (one subject) to account for possible dropouts.

### 2.9 Statistical analysis

The primary outcome of the study was set as the measurement of dental biofilm (TMQHPlI and VPI) on day 4 of each experimental arm. Secondary outcomes included differences in MGI, as well as differences in detected bacterial loads. All numerical variables were analyzed as means with standard deviations (SD) per subject per treatment. The normality of distribution was assessed using the Shapiro–Wilk test.

The analysis of variance (ANOVA) was used to determine any differences in TMQHPlI at the level of DDP and TDP between groups; patient and order of sequence were covariables. Tukey's honest significant difference tests were used as *post hoc* tests. Pearson's correlation coefficient between the level of DDP and TDP was calculated for each treatment modality.

The differences in VPI between all three mouthwashes were assessed using linear mixed models with random intercepts. The independent variables were: mouthwash type, mouthwash sequence, and the interaction between the two. The biofilm volume (VPI) was set as the dependent variable. Paired *post hoc* comparisons between individual mouthwashes were performed using Sidak tests. Pearson's correlation coefficient was used to calculate the correlation between the mean TMQHPlI and the mean VPI according to mouthwash type and tooth surface (oral/vestibular).

Microbiological outcomes were analyzed using linear mixed models, with patient and rinsing sequence as random factors, and the rinse type as a fixed factor, with respect to the baseline microbial load as a covariate. Contrasts were calculated using fixed factor estimates from the linear effects model and their variance‐covariance matrix. aMMP‐8 concentrations were analyzed using a censored normal (Tobit) regression model, with left‐censoring at 20 ng/mL (LOD of ORALyzer). The model included treatment, time, and their interaction as fixed effects. The reported means represent model‐based latent means. Wald contrasts were subsequently computed for pairwise comparisons. Statistical analyses were performed using SPSS v. 28 (IBM Statistics, Armonk, NY, USA); the significance threshold was set at *α* = 0.05.

## 3 RESULTS

### 3.1 Study population

Sixteen of 21 screened volunteers qualified for inclusion. All subjects but one (dropped out during the first study arm for personal reasons) completed all three arms of the study. The final sample comprised seven males and eight females, aged 22–30 years (mean age: 25.25 years). The CONSORT flow diagram of the study is presented in Figure S2.

### 3.2 Clinical outcomes

The full mouth and categorical TMQHPlI scores 4 days after rinsing with EOS, CHX, and placebo are shown in Table 1. The biofilm‐inhibitory effect of both CHX and EOS was significantly greater than that of placebo, with the effect of CHX being superior to that of EOS. A tendency towards higher TMQHPlI values (3, 4, 5) was found at interdental, vestibular, and maxillary sites.

**TABLE 1:** Mean TMQHPlI scores and standard deviations after 4 days according to mouthwash type.

	Placebo (*n* = 15) [mean (SD)]	EOS (*n* = 15) [mean (SD)]	CHX (*n* = 15) [mean (SD)]	ANCOVA *p*‐value	Placebo vs. EOS (*p*‐value)	Placebo vs. CHX (*p*‐value)	EOS vs. CHX (*p*‐value)
Full mouth							
TDP	2.49 (0.31)	2.27 (0.34)	1.63 (0.28)	**<0.001**	**0.033**	**<0.001**	**<0.001**
DDP	0.82 (0.42)	0.65 (0.36)	0.56 (0.20)	**0.026**	0.214	**0.040**	0.670
Non‐interdental sites							
TDP	2.45 (0.34)	2.18 (0.37)	1.44 (0.37)	**<0.001**	**0.032**	**<0.001**	**<0.001**
DDP	0.78 (0.43)	0.60 (0.37)	0.49 (0.18)	**0.025**	0.225	**0.027**	0.547
Interdental sites							
TDP	2.51 (0.31)	2.31 (0.34)	1.73 (0.26)	**<0.001**	**0.042**	**<0.001**	**<0.001**
DDP	0.85 (0.42)	0.67 (0.37)	0.59 (0.22)	**0.031**	0.228	0.050	0.746
Vestibular sites							
TDP	2.97 (0.48)	2.79 (0.39)	1.83 (0.37)	**<0.001**	0.306	**<0.001**	**<0.001**
DDP	1.23 (0.56)	0.93 (0.53)	0.80 (0.27)	**0.039**	0.161	**0.028**	0.674
Oral sites							
TDP	2.01 (0.25)	1.75 (0.45)	1.44 (0.26)	**<0.001**	**0.024**	**<0.001**	**0.008**
DDP	0.42 (0.39)	0.36 (0.29)	0.31 (0.15)	0.066	NA	NA	NA
Non‐molars							
TDP	2.47 (0.34)	2.23 (0.40)	1.63 (0.31)	**<0.001**	**0.036**	**<0.001**	**<0.001**
DDP	0.82 (0.47)	0.67 (0.43)	0.53 (0.22)	**0.019**	0.405	0.052	0.488
Molars							
TDP	2.53 (0.38)	2.35 (0.33)	1.64 (0.30)	**<0.001**	0.108	**<0.001**	**<0.001**
DDP	0.84 (0.38)	0.59 (0.34)	0.62 (0.25)	0.172	NA	NA	NA
Maxilla							
TDP	2.69 (0.31)	2.45 (0.35)	1.71 (0.33)	**<0.001**	**0.022**	**<0.001**	**<0.001**
DDP	1.18 (0.54)	0.92 (0.53)	0.75 (0.25)	**0.011**	0.141	0.007	0.375
Mandible							
TDP	2.29 (0.38)	2.09 (0.39)	1.55 (0.28)	**<0.001**	0.130	**<0.001**	**<0.001**
DDP	0.47 (0.42)	0.37 (0.29)	0.37 (0.19)	0.321^a^	NA	NA	NA

Abbreviations: EOS, electrolyzed saline mouthwash; CHX, chlorhexidine digluconate. mouthwash; SD, standard deviation; TDP, total disclosed plaque; DDP, dark colored disclosed plaque; NA, not applicable.

^a^ Logarithmic transformation of variables was performed during statistical analysis; bold values indicate statistically significant *p*‐values.

Pearson's correlation coefficients between the level of DDP and TDP according to treatment modality is presented in Table 2. A statistically significant correlation between DDP and TDP was observed only for chlorhexidine and was found to be moderate.

**TABLE 2:** Pearson's correlation coefficients between the level of DDP and TDP according to treatment modality.

Mouthwash	Pearson's *r* (95% confidence interval)	*p*‐value
Placebo	0.29 [−0.25 to 0.70]	0.280
EOS	0.41 [−0.13 to 0.76]	0.132
CHX	0.65 [0.21 to 0.87]	**0.009**

Abbreviations: EOS, electrolyzed saline mouthwash; CHX, chlorhexidine glucoronate mouthwash; bold values indicate statistically significant *p*‐values.

Baseline full mouth MGI levels were similar in all groups, ranging from 0.06 to 0.08. No statistically significant differences in GI were observed at follow‐up (Table S4).

### 3.3 Digital volumetric outcomes

**FIGURE 1:**
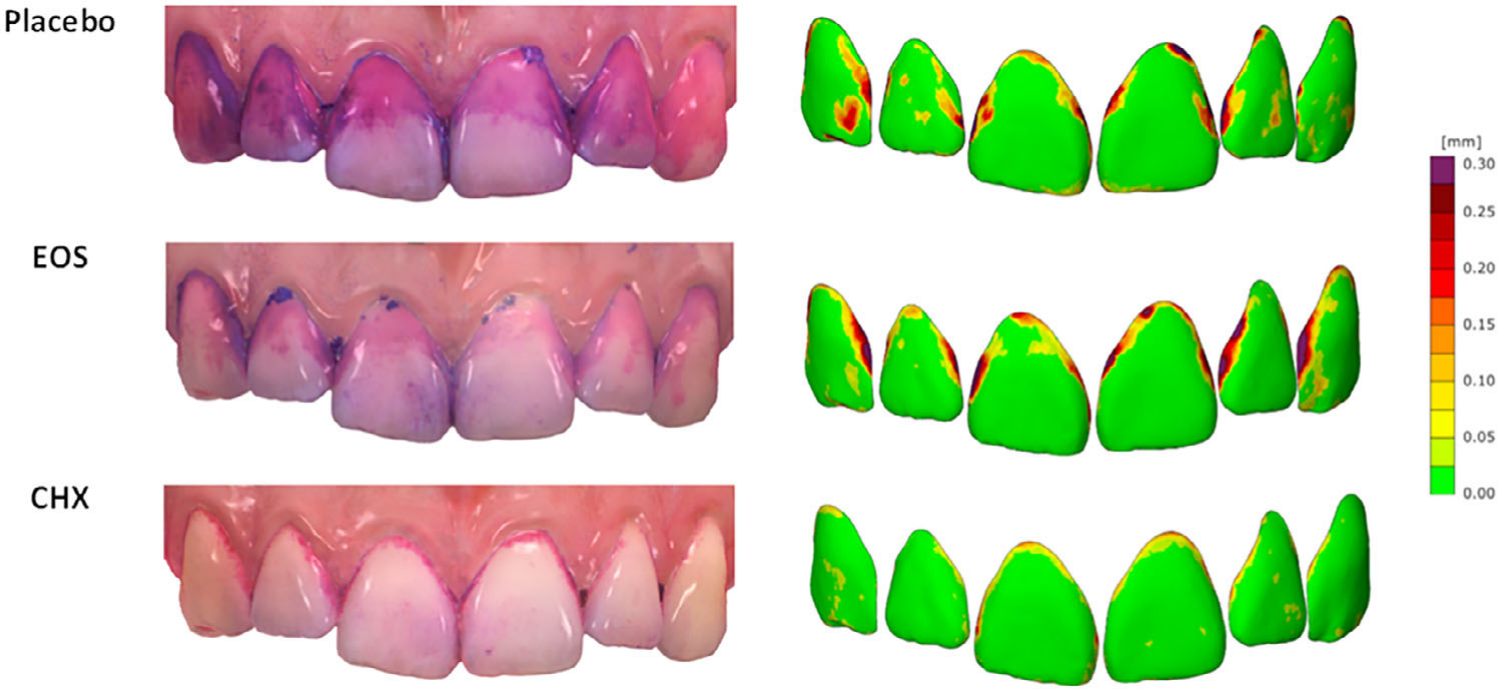
Digital evaluation of dental plaque 4 days after non‐brushing. Volumetric quantification of dental plaque using color maps (left) and screenshots from intraoral scans (right) for placebo, electrolyzed saline (EOS), and chlorhexidine digluconate (CHX).

The highest full‐mouth VPI score was found for placebo, followed by EOS and CHX (Figure 1). The biofilm‐inhibitory effect of CHX was significantly greater than that of both placebo and EOS. No difference was found between EOS and placebo (*p* > 0.05). Similar outcomes were observed at the level of vestibular sites, both the upper and lower jaw, as well as incisors, canines and molars (Table 3), as well as for AVPI scores (Table S5).

**TABLE 3:** Mean dental plaque volume (volumetric plaque index – VPI; mm^3^) and standard deviations after 4 days according to mouthwash type.

	Placebo (*n* = 15) [mean (SD)]	EOS (*n* = 15) [mean (SD)]	CHX (*n* = 15) [mean (SD)]	*p*‐value	Placebo vs. EOS (*p*‐value)	Placebo vs. CHX (*p*‐value)	EOS vs. CHX (*p*‐value)
Full mouth	0.78 (0.52)	0.73 (0.54)	0.55 (0.51)	**<0.001**	0.849	**<0.001**	**<0.001**
Site							
Vestibular	0.94 (0.48)	0.88 (0.49)	0.61 (0.45)	**<0.001**	0.998	**<0.001**	**<0.001**
Oral	0.60 (0.49)	0.57 (0.54)	0.50 (0.57)	0.463	0.762	0.530	0.961
Jaw							
Mandible	0.68 (0.44)	0.65 (0.44)	0.44 (0.28)	**<0.001**	0.97	**<0.001**	**<0.001**
Maxilla	0.87 (0.57)	0.81 (0.61)	0.67 (0.65)	**<0.001**	0.853	**<0.001**	**0.003**
Tooth type							
Incisor	0.75 (0.57)	0.70 (0.54)	0.51 (0.54)	**<0.001**	0.249	**<0.001**	**<0.001**
Canine	0.88 (0.43)	0.86 (0.75)	0.54 (0.39)	**<0.001**	0.956	**<0.001**	**<0.001**
Premolar	0.62 (0.41)	0.56 (0.36)	0.44 (0.43)	**0.017**	0.912	**0.024**	0.068
Molar	0.90 (0.55)	0.87 (0.51)	0.70 (0.58)	**0.003**	0.942	**0.035**	**0.004**

Abbreviations: EOS, electrolyzed saline mouthwash; CHX, chlorhexidine glucoronate mouthwash; SD, standard deviation; bold values indicate statistically significant *p*‐values.

Pearson's correlation coefficient showed a moderate relationship between both the traditional (DDP, TDP) and volumetric (VPI, AVPI) plaque indexes (Table 4).

**TABLE 4:** Pearson's correlation coefficient between traditional and volumetric plaque index scores.

	Turesky modification of the Quigley–Hein plaque index	
	TDP	DDP
VPI	0.63^a^	0.55^a^
AVPI	0.53^a^	0.56^a^

Abbreviations: VPI, volumetric plaque index; AVPI, adjusted volumetric plaque index; TDP, total disclosed plaque; DDP, dark colored disclosed plaque.

^a^ Statistically significant relationship (*p* < 0.001).

### 3.4 Microbiological and aMMP‐8 outcomes

At baseline (T0), the predominant species were the primary colonizers, including streptococci and anaerobic commensals. Additionally, *Treponema denticola*, a “red complex” bacteria, and *Fusobacterium nucleatum*, were present.

The placebo mouthwash evoked a normal biofilm development pattern, leading to an overall increase in bacterial load and a proliferation of all selected key‐species, with a dominance of anaerobic commensals.

**FIGURE 2:**
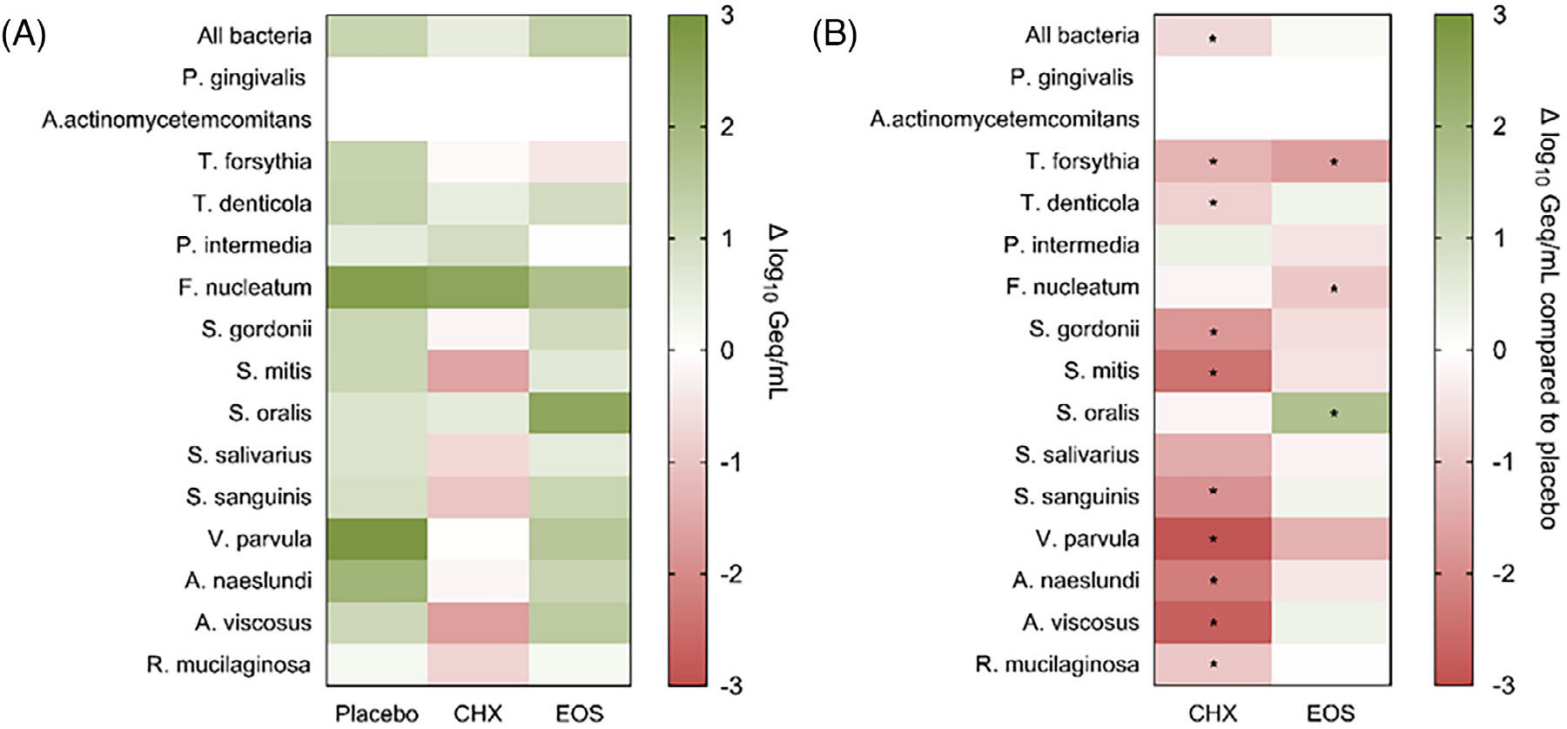
(A) Increases (green) and decreases (red) in selected oral species 4 days after rinsing with placebo, chlorhexidine (CHX), and electrolyzed saline (EOS), compared with baseline. (B) Increases (green) and decreases (red) in selected oral species 4 days after rinsing with CHX and EOS, compared with placebo. Note: Placebo, distilled water; EOS, electrolyzed saline mouthwash; CHX, chlorhexidine digluconate mouthwash; *statistically significant *p*‐values for mean difference with reference to placebo; Geq, genomic equivalent.

Chlorhexidine exhibited the most notable antimicrobial activity over a 4‐day period, reducing the total bacterial burden by ≈0.7 log_10_ (≈80%) compared with placebo, its non‐specific antimicrobial activity primarily affected selected commensals. The most significant was the reduction in the mitis group of streptococci (*Streptococcus mitis, Streptococcus gordonii*, and *Streptococcus sanguinis*). Although *Streptococcus salivarius* and *Streptococcus oralis* also showed a decline, the reduction was not statistically significant. CHX further exhibited antimicrobial effects against periodontal health‐associated *Rhodotorula mucilaginosa* and the anaerobic commensals *Actinomyces viscosus, Actinomyces naeslundii*, and *V. parvula*. In contrast, EOS only affected some of the measured key‐species periodontal pathobionts while preserving commensals; it did not alter the total bacterial burden and notably increased the abundance of *S. oralis* (Figure 2a and b).

Since only periodontally healthy subjects were included in the trial, the putative periodontal pathobionts *Porphyromonas gingivalis* and *Aggregatibacter actinomycetemcomitans* were absent in all individuals. Substantial reductions in the load of *Tannerella forsythia* were observed after rinsing with both CHX and EOS. Only EOS decreased the levels of *F. nucleatum* and *Prevotella intermedia* compared with placebo, however, the reduction of the latter was not statistically significant. When compared with CHX, EOS consistently demonstrated a stronger antimicrobial effect against all selected periodontal pathobionts, except for *T. denticola*. The subgingival microbiological data are presented in Table S6.

The levels of a‐MMP‐8 were below 20 ng/mL in >80% of subjects at both T0 and T4, with no statistically significant differences between the two timepoints or among the different mouthwashes (Table S7).

## 4 DISCUSSION

The aim of the study was to evaluate the biofilm‐inhibitory and microbiological effects of EOS in a 4‐day non‐brushing model compared with positive (CHX) and negative controls. Both the VPI and TMQHPlI showed that the total effect of CHX on biofilm control was superior to EOS, even though both significantly reduced the biofilm levels compared with placebo. However, the non‐selective broad‐spectrum antimicrobial effect of CHX led to significant reductions in subgingival bacterial loads of all selected key‐species commensals and caused an unfavorable alteration of the biofilm composition. The microbiological effect of EOS was selective. It significantly reduced the growth of some periodontal pathobionts and had no significant effect on primary‐colonizing, periodontal health‐associated bacteria.

Chlorine‐based antimicrobials are pH‐dependent solutions whose composition, mode of action, antimicrobial efficacy, and (cyto)toxicity depend on the balance between hypochlorite (OCl^¯^) (alkaline) and hypochlorous acid (HOCl) (neutral/acidic).^21^ Chlorine‐based solutions are used in dentistry as surface disinfectants, endodontic rinses, wound antiseptics, and dental coolants.^8^, ^22^ In addition, the American Dental Association Council on Dental Therapeutics has designated 0.1% NaOCl as a “mild antiseptic mouth rinse” and recommended its use for direct application to mucous membranes.^23^ However, clinical studies on NaOCl mouthwashes show heterogeneity in methodology due to different study designs, inclusion criteria, application regimens, concentrations of chlorine compounds, and frequency/duration of rinsing.^24^ A systematic review^24^ found that studies with a negative control group provided weak evidence of a small beneficial effect of NaOCl mouthwashes on PlI, GI, and BOP, while studies with a positive control provided weak evidence that NaOCl mouthwashes had a similar effect to CHX on PlI, GI, and BOP.

These notions are consistent with the TMQHPlI results of the present study, whereby EOS only significantly reduced the full‐mouth TDP (by 9%), while CHX significantly reduced both the TDP (35%) and DDP (32%) compared with placebo, and demonstrated stronger biofilm‐inhibitory effects than EOS in all TDP categories. EOS appears more effective against areas with a lower plaque density (contributing to overall TDP reduction) but was less effective against the densely packed microbial communities that form DDP, even within the short 4‐day timeframe. Research shows that biofilms can develop structural complexity within 48–72 h, with increased cell density, enhanced extracellular matrix production, and the formation of microcolonies in localized zones of higher bacterial density.^25^ The lack of statistical significance for DDP may also reflect reduced statistical power due to the inherently lower DDP baseline values (TMQHPlI = 0.65–0.82) compared with TDP values (TMQHPlI = 2.27–2.49). Despite showing a 20.7% reduction in DDP versus 8.8% in TDP, the higher variability and smaller absolute differences in the DDP measurements make it harder to achieve statistical significance with the limited sample size (*n* = 15).

Although traditional plaque indices^11^, ^26^, ^27^ remain the gold standard for biofilm assessment, they are subjective and only assess the distribution/location of biofilms, even in cases with two‐color disclosing solutions that provide thickness‐dependent differential staining, that is, pink‐dye (putative “new biofilm”) and blue‐dye (putative “mature/old” biofilm).^28^, ^29^, ^30^ To the best of our knowledge, no study to date has used a digital biofilm‐scoring approach based on superimposed serial 3D IOSs to evaluate the effect of mouthwashes on biofilm volume changes. The main advantages of volumetry are objectivity, higher sensitivity, non‐invasiveness, and the absence of subjectivity/variability of the examiner. This is particularly important at gingival margins, where biofilms are most prevalent and often uneven due to site‐specific variables, for example, tooth anatomy. Color‐coded maps from different time‐points can also be compared to show biofilm predilection sites and to serve as a tool to motivate oral hygiene.^15^ The digital volumetric analysis showed that while EOS had a significant biofilm‐inhibiting effect, it was still inferior to CHX. CHX reduced the full‐mouth VPI by 29%, while EOS reduced it by 6% compared with placebo (0.78 mm^3^). Thus, both the traditional and digital biofilm‐scoring approaches confirmed the clinical superiority of CHX.^14^, ^31^ The biofilm‐inhibitory effect of CHX was further enhanced by the design of the present study, in which all subjects were instructed to rinse twice‐daily (after breakfast and dinner) with CHX, as this interval corresponds to the 12‐h sustained release of CHX.^32^ The antiplaque effect may be further improved by delaying CHX rinsing for at least 30 min after toothbrushing, as longer intervals reduce inactivation by toothpaste ingredients.^7^ The volumetric evaluation, nevertheless, had its limitations due to scanning distortions, especially in posterior teeth, curved dental arches, and interdental spaces, which may reduce superimposition accuracy.^12^ In addition, the relevance of the approach needs to be assessed in future trials.

Although CHX exhibited the strongest biofilm‐inhibitory properties and decreased the total microbial loads, it had a non‐selective control effect on both early colonizers and periodontopathogens. In agreement with other studies, even short‐term CHX treatment can have a negative impact on nitrate‐reducing bacteria (*A. naeslundi, A. viscosus, R. mucilaginosa*, *V. parvula*) and can impair the bacterial capacity of nitrate–nitrite–nitric oxide metabolisms.^33^, ^34^, ^35^ The results of our study argue against the uncritical use of CHX in clinical practice, with regard to its controversial role in ecological microbial changes^8^, ^36^ and its tendency to promote antibiotic cross‐resistance.^37^, ^38^

Conversely, EOS showed a potential dysbiosis‐controlling effect. Its selective antimicrobial activity affected *F. nucleatum*, a key bridge bacteria facilitating biofilm maturation, and *T. forsythia*, while the measured key commensals were spared. This effect could potentially delay dysbiotic plaque development. Similar dysbiosis‐preventing results were found in an in vitro multispecies model of subgingival biofilms, where rinsing with EOS reduced major pathobionts but did not affect oral commensals or human oral keratinocytes.^8^

Selective recolonization, and consequently delayed biofilm maturation, are difficult to confirm in periodontally healthy subjects, who harbor a less complex microbiota than patients with periodontitis,^38^ over a 4‐day observation period. HOCl acts as a non‐specific oxidizing agent, and introducing such oxidative conditions to an early biofilm likely perturbs its natural development which is generally accompanied by a gradual shift toward anaerobiosis and an increased prevalence of Gram‐negative species. However, the precise mechanism of HOCl action within the biofilm remains speculative. We assume that a change in biofilm niche, driven by high redox potential resulting in oxidative stress,^39^ together with the altered influx of nutrients associated with necrotrophy,^40^ favors the predominance of commensal streptococci, which exhibit higher growth rates than strict anaerobes, possibly because the latter depend on an established metabolic network and biofilm succession toward anaerobic conditions. Thus, recolonization appears to be primarily driven by biofilm dynamics.

The more pronounced action of EOS against periodontopathogens may be related to their Gram‐negative cell structure, as these species are more susceptible to irreversible HOCl‐induced damage due to the sulfur‐ and heme‐containing membrane components essential for adhesion.^41^, ^42^ Such membrane damages^43^ may reduce their colonization potential^44^ and contribute to the enhanced resilience of the biofilm against dysbiosis, as observed after rinsing with EOS. However, to confirm these, further in vitro studies investigating the underlying mechanisms, as well as clinical trials involving periodontitis patients, are required. Nevertheless, our findings indicate that EOS had no adverse effect on the early subgingival microbiota composition.

Similarly, an analysis of the salivary microbiome of healthy patients revealed that *ex‐vivo* exposure to HOCl can inhibit or delay the formation of oral biofilms while maintaining the overall balance of the oral microbiome; the only bacterial genus that showed a significant decrease was *Porphyromonas*.^45^ These results suggest a potential positive clinical effect of HOCl‐containing solutions in the maintenance of biofilm resilience and supports its efficacy for clinical use as a mouthrinse.^46^

The present study employed an established 4‐day plaque‐accumulation model to evaluate the antibacterial and biofilm‐inhibiting effects of oral hygiene products,^13^ as most biofilm formation occurs within the first 4 days without tooth brushing, followed by only moderate additional accumulation.^47^ However, this short duration may not fully capture the dynamic maturation processes of the oral microbiota during continued biofilm development. Furthermore, the study population consisted of periodontally healthy individuals with a relatively low baseline subgingival load of periodontal pathobionts, both of which are limitations of the study. The inclusion of a placebo arm nevertheless provided a true baseline for assessing the efficacy of EOS and CHX, enabling clear differentiation between the effects of the active interventions and natural plaque regrowth. In addition, the study design and statistical analysis accounted for the sequence of mouthwashes received by each participant, which, in addition to the 7‐day washout period, helped to control for potential residual effects and minimized their impact on the observed outcomes.

The non‐significant GI and aMMP‐8 outcomes were most likely a result of the short observation period, in addition to the fact that subjects with periodontal pathologies/signs of gingivitis were excluded. The absence of significant aMMP‐8 changes likely reflects the absence of neutrophil‐driven inflammatory pathways and their contribution to biofilm composition. Neutrophils, the primary source of aMMP‐8, release extracellular traps (NETs)^48^ that integrate into the biofilm, alter its structure, and influence bacterial composition.^49^ The low aMMP‐8 levels observed suggest minimal neutrophil involvement, indicating that the study primarily captured the direct effects of antimicrobial mouthrinses on pure bacterial biofilms. Future studies should focus on measuring rapidly changing inflammatory mediators such as IL‐2, IL‐6, and TNF‐α to capture early subclinical responses.^50^

A limitation of this study is that supragingival plaque sampling and microbiota profiling were not performed, which would have clarified the (early) ecological effects of the tested antimicrobials. However, by analyzing key oral bacterial species, this study offers a representative framework for assessing how different mouthwashes influence the subgingival microbial community. Another limitation was the choice of native active ingredients, which resulted in poor organoleptic properties of the mouthwashes. Even though the most frequently reported side effect of EOS was the unpleasant taste immediately after rinsing, this could have been minimized by using a taste enhancer.

## 5 CONCLUSIONS

The effect of chlorhexidine on biofilm control was superior to that of EOS at the level of TMQHPlI and VPI, even though both substantially reduced biofilm levels compared with placebo. A selective microbiologic effect of EOS was observed. EOS primarily targeted periodontal pathobionts, while commensal bacteria were preserved. In contrast, CHX had a robust effect in preventing the regrowth of not only pathobionts but also commensals.

## AUTHOR CONTRIBUTIONS

*Conception and design*: Katja Povšič, Haris Munjaković, Rok Gašperšič, Wim Teughels. *Data collection*: Katja Povšič, Haris Munjaković, Naiera Zayed. *Data analysis*: Katja Povšič, Haris Munjaković, Naiera Zayed, Rok Gašperšič. *Data interpretation*: Katja Povšič, Haris Munjaković, Naiera Zayed, Katja Seme, Aleš Fidler, Rok Gašperšič. *Drafting of manuscript*: Katja Povšič, Haris Munjaković, Naiera Zayed, Rok Gašperšič. *Critical revision of manuscript*: Katja Povšič, Haris Munjaković, Rok Gašperšič, Naiera Zayed, Katja Seme, Aleš Fidler, Wim Teughels. All authors have given final approval of the version to be published and agree to be accountable for all aspects of the work in ensuring that questions related to the accuracy or integrity of any part of the work are appropriately investigated and resolved.

## CONFLICT OF INTEREST STATEMENT

The authors declare no potential conflicts of interest with respect to the research, authorship, and/or publication of this article.

## Supporting information



Supporting Information

## Data Availability

The data that support the findings of this study are available upon request from the corresponding author. The data are not publicly available due to privacy or ethical restrictions.
