# Supplementary material for: Electrolyzed saline as an alternative to chlorhexidine: Antimicrobial and biofilm volume outcomes in a 4‐day non‐brushing randomized controlled clinical trial
Source: J Periodontol. 2026 Feb 2;97(6):1231–42. doi: 10.1002/jper.70070 (PMC13350408; doi:10.1002/jper.70070)
Supplement: Supplementary file 1 — Supporting Information [file JPER-97-1231-s001.docx]

**SUPPLEMENTAL APPENDIX**

**Electrolyzed Saline as an Alternative to Chlorhexidine: Antimicrobial and Biofilm Volume Outcomes in a 4-Day Non-Brushing RCCT**

***Electrolyzed saline modulates plaque re-growth***

Katja Povšič (DMD)^*,†^, Haris Munjaković (MSCI) ^*,†^, Naiera Zayed (PhD)^‡ §^, Wim Teughels (PhD, DMD)^‡^, Katja Seme (PhD, MD) ^‖^, Aleš Fidler (PhD, MDM)^¶#^, Rok Gašperšič (PhD, DMD) ^*,†^

^*^ Department of Oral Medicine and Periodontology, Faculty of Medicine, University of Ljubljana, Ljubljana, Slovenia

^†^ Department of Oral Medicine and Periodontology, University Medical Centre Ljubljana, Ljubljana, Slovenia

^‡^ Department of Oral Health Sciences, University of Leuven (KU Leuven), Leuven, Belgium

^§^ Department of Microbiology and Immunology, Faculty of Pharmacy, Menoufia University, Menoufia, Egypt

^‖^ Institute of Microbiology and Immunology, Faculty of Medicine, University of Ljubljana, 1000 Ljubljana, Slovenia

^¶^ Department of Endodontics and Operative Dentistry, Faculty of Medicine, University of Ljubljana, Ljubljana, Slovenia

^#^ Department of Restorative Dentistry and Endodontics, University Medical Centre Ljubljana, Ljubljana, Slovenia

**Corresponding author:** Katja Povšič, Department of Oral Medicine and Periodontology, Faculty of Medicine, University of Ljubljana, Hrvatski trg 6, 1000 Ljubljana, Slovenia; telephone: 0038615224889 (katja.povsic@kclj.si).

**Supplementary table 1.** Inclusion and exclusion criteria.

| **Inclusion criteria** | **Exclusion criteria** |
| --- | --- |
| - Age 20-30 years - Non-smokers - Systemically healthy - No regular systemic medication - Presence of at least 6 teeth in each jaw quadrant - Turesky modification of Quigley Hein Plaque Index < 10 % at baseline - No periodontal pockets measuring > 4 mm - Interdental clinical attachment loss (CAL) is not detectable at any tooth - Buccal or oral CAL ≥3 mm with pocketing >3 mm is not detectable at any tooth | - Gingivitis - Regular use of a mouthwash as part of oral hygiene routine - Known allergy or suspected hypersensitivity to chlorhexidine digluconate - Antibiotic treatment in the last 6 months - Pregnancy - Lactation - Fixed/removable prosthetic devices - Dental implants - Systemic illness (i.e. HIV/AIDS, diabetes mellitus, cancer, bone metabolism diseases, diseases affecting wound healing) - Treatment with immunosuppressive therapy, chemotherapy, radiation therapy, calcium antagonists, antiepileptics, non-steroidal anti-inflammatory drugs |

**Supplementary table 2.** Key oral microbial species selected for detection and quantification in the subgingival plaque samples.

| **Bacterial complex** | **Bacterial species** |
| --- | --- |
| **Periodontal-disease associated bacteria** | Prevotella intermedia  Porphyromonas gingivalis  Fusobacterium nucleatum  Aggregatibacter actinomycetemcomitans  Treponema denticola  Tannerella forsythia |
| **Periodontal-health associated bacteria** | Rothia mucilaginosa  Streptococcus sanguinis  Actinomyces viscosus  Actinomyces naeslundii |
| **Core microbiome bacteria** | Veillonella parvula  Streptococcus gordonii  Streptococcus oralis  Streptococcus mitis  Streptococcus salivarius |

| **Species** | **Species-specific primers** | |
| --- | --- | --- |
| All bacteria | Forward | CGC CGG GGG CGC GCC CCG GGC GGG GCG GGG GCA CGG GGG GAC TCC TAC GGG AGG CAG CAG |
|  | Reverse | ATT ACC GCG GCT GG |
| *Aggregatibacter actinomycetemcomitans* | Forward | CGG TGT CGA TTT GGG GAT TGG |
|  | Reverse | TGC AGC ACC TGT CTC AAA GC |
| *Prevotella intermedia* | Forward | TGT GCC CYT TTG CAT TTA CCC TTC |
|  | Reverse | CAC CAT GAA TTC CGC ATA CG |
| *Porphyromonas gingivalis* | Forward | CCG TAA GAA TAA GCA TCG GCT AAC TC |
|  | Reverse | CAC GAA TTC CGC CTG C |
| *Fusobacterium nucleatum* | Forward | GGA TTT ATT GGG CGT AAA GC |
|  | Reverse | ATC TGT CCA GTA AGC TGG CTT CC |
| *Treponema denticola* | Forward | GGT AAA TGA GGA AAG GAG CTA CGG C |
|  | Reverse | GGA TAC CCA TCGC CT TGG T |
| *Tannerella forsythia* | Forward | GGG TGA GTA ACG CGT ATG TAA CCT |
|  | Reverse | ACC CAT CCG CAA CCA ATA AA |
| *Actinomyces naeslundii* | Forward | TCG AAA CTC AGC AAG TAG CCG |
|  | Reverse | AGA GGA GGG CCA CAA AAG AAA |
| *Actinomyces viscosus* | Forward | GTG AAG GAG CCA GCT TGC TGG TTC TG |
|  | Reverse | CGG AAC AAA CCT TTC CCA GGC |
| *Veillonella parvula* | Forward | GAC GAA AGT CTG ACG GAG CA |
|  | Reverse | TGC CAC CTA CGT ATT ACC GC |
| *Streptococcus oralis* | Forward | ACC AGC AGA TAC GAA AGA AGC AT |
|  | Reverse | AGG TTC GGG CAA GCG ATC TTT CT |
| *Streptococcus sanguinis* | Forward | CAA AAT TGT TGC AAA TCC AAA GG |
|  | Reverse | GCT ATC GCT CCC TGT CTT TGA |
| *Streptococcus gordonii* | Forward | GAA GAA CTG GGT AGC GAT TGC T |
|  | Reverse | GTT AGC TGT TGG ATT GGT TGC C |
| *Streptococcus mitis* | Forward | GGC TCG TAG TCT GGA GAT GG |
|  | Reverse | TAG GTC GTC GTC CCA AGG AA |
| *Streptococcus salivarius* | Forward | GAC GAT GAC TGT CAA CTT GAC AC |
|  | Reverse | ACC GTA ACG TGG GAA AAC TG |
| *Rothia mucilaginosa* | Forward  Reverse | ACA CCA TYA AGT ACT ACG G  TAC CAG TCG TAG AAG CTG |

**Supplementary table 3.** Primers list for v-qPCR detection of selected key bacterial species**.**

**Supplementary table 4**. Mean MGI scores and standard deviations after 4 days. The three groups were compared by means of ANCOVA (with patient, order of sequence and baseline MGI as covariables).

|  | **Placebo (n = 15)**  **[mean (SD)]** | **EOS (n = 15)**  **[mean (SD)]** | **CHX  (n = 15)**  **[mean (SD)]** | **p-value** |
| --- | --- | --- | --- | --- |
| **Full mouth** |  |  |  |  |
| Baseline MGI | 0.06 (0.05) | 0.08 (0.09) | 0.07 (0.08) |  |
| Follow-up MGI | 0.10 (0.08) | 0.08 (0.11) | 0.09 (0.11) | 0.271 |
| **Non-interdental sites** |  |  |  |  |
| Baseline MGI | 0.09 (0.07) | 0.11 (0.12) | 0.10 (0.11) |  |
| Follow-up MGI | 0.14 (0.10) | 0.09 (0.12) | 0.12 (0.15) | 0.161 |
| **Interdental sites** |  |  |  |  |
| Baseline MGI | 0.03 (0.04) | 0.05 (0.07) | 0.04 (0.06) |  |
| Follow-up MGI | 0.06 (0.07) | 0.06 (0.10) | 0.06 (0.06) | 0.969 |
| **Vestibular sites*** |  |  |  |  |
| Baseline MGI | 0.05 (0.06) | 0.07 (0.10) | 0.06 (0.08) |  |
| Follow-up MGI | 0.07 (0.08) | 0.05 (0.08) | 0.06 (0.10) | 0.475 |
| **Oral sites*** |  |  |  |  |
| Baseline MGI | 0.08 (0.07) | 0.10 (0.12) | 0.09 (0.12) |  |
| Follow-up MGI | 0.13 (0.11) | 0.10 (0.14) | 0.12 (0.13) | 0.258 |
| **Non-molars*** |  |  |  |  |
| Baseline MGI | 0.03 (0.04) | 0.06 (0.07) | 0.05 (0.06) |  |
| Follow-up MGI | 0.06 (0.06) | 0.04 (0.06) | 0.06 (0.11) | 0.376 |
| **Molars** |  |  |  |  |
| Baseline MGI | 0.13 (0.15) | 0.12 (0.16) | 0.13 (0.18) |  |
| Follow-up MGI | 0.19 (0.18) | 0.16 (0.23) | 0.17 (0.16) | 0.835 |
| **Maxilla** |  |  |  |  |
| Baseline MGI | 0.08 (0.07) | 0.09 (0.09) | 0.07 (0.06) |  |
| Follow-up MGI | 0.12 (0.10) | 0.08 (0.10) | 0.10 (0.10) | 0.214 |
| **Mandible*** |  |  |  |  |
| Baseline MGI | 0.05 (0.06) | 0.08 (0.10) | 0.08 (0.11) |  |
| Follow-up MGI | 0.08 (0.08) | 0.07 (0.12) | 0.08 (0.13) | 0.507 |

MGI – modified gingival index; EOS – electrolyzed saline mouthwash; CHX – chlorhexidine glucoronate mouthwash; SD – standard deviation; * – logarithmic transformation of variables was performed during statistical analysis, but did not normalize the distribution of values.

**Supplementary table 5.** Mean adjusted dental plaque volume (adjusted volumetric plaque index – AVPI; mm^3^/mm^2^) and standard deviations after 4 days according to mouthwash type.

|  | **Placebo (n = 15) [mean (SD)]** | **EOS (n = 15) [mean (SD)]** | **CHX  (n = 15) [mean (SD)]** | **p-value** | **Placebo vs. EOS (p-value)** | **Placebo vs. CHX (p-value)** | **EOS vs. CHX (p-value)** |
| --- | --- | --- | --- | --- | --- | --- | --- |
| **Full mouth** | 0.0169 (0.0115) | 0.0159 (0.0117) | 0.0119 (0.0101) | **< 0.001** | 0.674 | **< 0.001** | **< 0.001** |
| **Site** |  |  |  |  |  |  |  |
| Vestibular | 0.0178 (0.0103) | 0.0165 (0.0091) | 0.0111 (0.0073) | **< 0.001** | 0.909 | **< 0.001** | **< 0.001** |
| Oral | 0.0159 (0.0)125 | 0.0152 (0.0)139 | 0.0129 (0.0123) | 0.181 | 0.778 | 0.187 | 0.586 |
| **Jaw** |  |  |  |  |  |  |  |
| Mandible | 0.0170 (0.0122) | 0.0159 (0.0)106 | 0.0105 (0.0058) | **< 0.001** | 0.550 | **< 0.001** | **< 0.001** |
| Maxilla | 0.0168 (0.0107) | 0.0159 (0.0127) | 0.0134 (0.0128) | **0.008** | 0.970 | **0.016** | **0.025** |
| **Tooth type** |  |  |  |  |  |  |  |
| Incisor | 0.0178 (0.0148) | 0.0165 (0.0136) | 0.0125 (0.0120) | **< 0.001** | 0.991 | **< 0.001** | **< 0.001** |
| Canine | 0.0178 (0.0107) | 0.0182 (0.0174) | 0.0109 (0.0074) | **< 0.001** | 0.929 | **< 0.001** | **< 0.001** |
| Premolar | 0.0166 (0.0106) | 0.0151 (0.0084) | 0.0120 (0.0104) | **0.024** | 0.974 | **0.041** | 0.066 |
| Molar | 0.0160 (0.0088) | 0.0155 (0.0081) | 0.0123 (0.0081) | **0.008** | 0.853 | **0.007** | 0.095 |

EOS – electrolyzed saline mouthwash; CHX – chlorhexidine glucoronate mouthwash; SD – standard deviation; bold values – statistically significant p-values.

**Supplementary table 6**. Microbiological outcomes.

|  | **Mouthwash** | **Frequency detected at baseline** | | **Frequency detected after intervention** | **Baseline mean (log_10_Geq/mL)** | **Mean difference:**  **baseline v. outcome (log_10_Geq/mL)** | **Mean difference in reference to placebo (log_10_Geq/mL)** | **95% CI of mean difference in reference to placebo** | **p-value** |
| --- | --- | --- | --- | --- | --- | --- | --- | --- | --- |
| **Total bacterial load** | CHX | 15/15 | | 15/15 | 6.712 | 0.520 | -0.670 | -1.163 – -0.319 | 0.010* |
|  | EOS | 15/15 | | 15/15 | 6.808 | 1.333 | 0.142 | - 0.342 – 0.627 | 0.511 |
|  | PLACEBO | 15/15 | | 15/15 | 6.902 | 1.190 | / | / |  |
| **Periodontal disease-associated bacteria** | | |  | | | | | | |
| ***Pg*** | CHX | 0/15 | | 0/15 | ND | ND | ND | ND | ND |
|  | EOS | 0/15 | | 0/15 | ND | ND | ND | ND | ND |
|  | PLACEBO | 0/15 | | 0/15 | ND | ND | ND | ND | ND |
| ***Aa*** | CHX | 0/15 | | 0/15 | ND | ND | ND | ND | ND |
|  | EOS | 0/15 | | 0/15 | ND | ND | ND | ND | ND |
|  | PLACEBO | 0/15 | | 0/15 | ND | ND | ND | ND | ND |
| ***Tf*** | CHX | 5/15 | | 4/15 | 0.906 | - 0.107 | -1.318 | -2.061 – -0.574 | 0.001* |
|  | EOS | 8/15 | | 4/15 | 1.765 | - 0.436 | -1.647 | -2.391 – -0.903 | < 0.001* |
|  | PLACEBO | 4/15 | | 7/15 | 0.826 | 1.211 | / | / | / |
| ***Td*** | CHX | 15/15 | | 15/15 | 5.466 | 0.473 | -0.793 | -1.436 – -0.150 | 0.018* |
|  | EOS | 15/15 | | 15/15 | 5.641 | 0.963 | 0.303 | -0.946 – 0.340 | 0.342 |
|  | PLACEBO | 15/15 | | 15/15 | 5.406 | 1.266 | / | / | / |
| ***Pi*** | CHX | 3/15 | | 4/15 | 0.291 | 0.948 | 0.396 | -0.901 – 1.692 | 0.537 |
|  | EOS | 2/15 | | 4/15 | 0.386 | 0.046 | -0.507 | -1.803 – 0.790 | 0.430 |
|  | PLACEBO | 3/15 | | 2/15 | 0.664 | 0.553 | / | / | / |
| ***Fn*** | CHX | 11/15 | | 15/15 | 3.544 | 2.480 | -0.202 | -1.058 – 0.655 | 0.642 |
|  | EOS | 14/15 | | 14/15 | 4.198 | 1.741 | -0.940 | -1.797 – -0.084 | 0.032* |
|  | PLACEBO | 11/15 | | 14/15 | 3.089 | 2.681 | / | / | / |
| **Core microbiome bacteria** | |  | |  |  |  |  |  |  |
| ***S. gordonii*** | CHX | 7/15 | | 7/15 | 2. 306 | -0.192 | -1.788 | -3.504 – -0.071 | 0.042* |
|  | EOS | 7/15 | | 10/15 | 2. 659 | 1.006 | -0.590 | -2.306 – 1.126 | 0.492 |
|  | PLACEBO | 7/15 | | 11/15 | 2. 605 | 1.596 | / | / | / |
| ***S. mitis*** | CHX | 12/15 | | 9/15 | 4.034 | -1.568 | -2.394 | -4.595 – -0.193 | 0.034* |
|  | EOS | 11/15 | | 12/15 | 3.987 | 0.625 | -0.504 | -2.702 – 1.700 | 0.645 |
|  | PLACEBO | 12/15 | | 14/15 | 4.263 | 1.126 | / | / | / |
| ***S. oralis*** | CHX | 14/15 | | 14/15 | 4.849 | 0.553 | -0.189 | -1.610 – 1.231 | 0.790 |
|  | EOS | 12/15 | | 15/15 | 4.365 | 2.450 | 1.707 | 0.286 – 3.128 | 0.020* |
|  | PLACEBO | 14/15 | | 14/15 | 5.220 | 0.743 | / | / | / |
| ***S. salivarius*** | CHX | 15/15 | | 15/15 | 1.102 | -0.665 | -1.429 | -3.121– 0.263 | 0.095 |
|  | EOS | 3/15 | | 5/15 | 0.711 | 0.533 | -0.212 | -1.904 – 1.480 | 0.800 |
|  | PLACEBO | 3/15 | | 6/15 | 0.785 | 0.764 | / | / | / |
| ***Vp*** | CHX | 12/15 | | 11/15 | 5.201 | -0.017 | -2.906 | -4.852 – -0.960 | 0.005* |
|  | EOS | 15/15 | | 15/15 | 6.324 | 1.563 | -1.327 | -3.273 – 0.619 | 0.174 |
|  | PLACEBO | 10/15 | | 14/15 | 4.531 | 2.889 | / | / | / |
| **Periodontal health-associated bacteria** | |  | |  |  |  |  |  |  |
| ***S. sanguinis*** | CHX | 5/15 | | 2/15 | 5.194 | -0.993 | -1.873 | -2.625 – -1.120 | < 0.001* |
|  | EOS | 3/15 | | 5/15 | 5.097 | 1.160 | 0.281 | -0.472 – 1.033 | 0.451 |
|  | PLACEBO | 7/15 | | 14/15 | 5.306 | 0.879 | / | / | / |
| ***An*** | CHX | 13/15 | | 12/15 | 5.239 | -0.174 | -2.226 | -3.955 – -0.497 | 0.005* |
|  | EOS | 15/15 | | 15/15 | 6.307 | 1.169 | -0.433 | -2.162 – 1.296 | 0.174 |
|  | PLACEBO | 13/15 | | 14/15 | 5.315 | 2.052 | / | / | / |
| ***Av*** | CHX | 13/15 | | 6/15 | 3.038 | -1.645 | -2.722 | -3.928 – -1.516 | < 0.001* |
|  | EOS | 9/15 | | 13/15 | 2.111 | 1.423 | 0.346 | -0.860 – 1.552 | 0.567 |
|  | PLACEBO | 10/15 | | 11/15 | 2.398 | 1.077 | / | / | / |
| ***Rm*** | CHX | 15/15 | | 15/15 | 4.031 | -0.755 | -0.960 | -1.325 – -0.576 | < 0.001* |
|  | EOS | 15/15 | | 15/15 | 3.892 | 0.185 | -0.02 | -0.384 – 0.344 | 0.913 |
|  | PLACEBO | 15/15 | | 15/15 | 3.937 | 0.205 | / | / | / |

EOS – electrolyzed saline mouthwash; CHX – chlorhexidine glucoronate mouthwash; SD – standard deviation; CI – confidence interval; * – statistically significant p-values for mean difference with reference to placebo; Geq – genomic equivalent; Pg – *Porphyromonas gingivalis*; Aa – *Aggregatibacter actinomycetemcomitans*; Tf – T*annerella forsythia*; Td – *Treponema denticola*; Pi – P*revotella intermedia*; Fn – Fusobacterium *nucleatum;* *S. gordonii* – *Steptococcus gordonii*; *S*. *mitis* – *Streptococcus mitis*; *S. oralis* – *Streptococcus oralis*; *S. sanguinis* – *Streptococcus sanguinis;* Vp – *Veillonella parvula*; An – *Actinomyces naeslundi*; Av – *Actinomyces viscosus*; Rm – *Rothia mucilaginosa*

**Supplementary table 7.** Tobit regression estimates for aMMP-8 levels: comparisons between treatments at baseline and day 4, and within-treatment changes.

|  | **aMMP-8 latent mean difference (ng/mL)^1^** | **p-value^2^** |
| --- | --- | --- |
| **Baseline (T0)** |  |  |
| CHX vs Placebo | 2.0 | 0.799 |
| CHX vs EOS | -0.5 | 0.952 |
| EOS vs Placebo | 2.5 | 0.745 |
| **After 4-days (T4)** |  |  |
| CHX vs Placebo | 11.0 | 0.272 |
| CHX vs EOS | 9.4 | 0.358 |
| EOS vs Placebo | 1.6 | 0.851 |
| **Within treatment changes (T4-T0)** |  |  |
| Placebo | -4.3 | 0.583 |
| EOS | -3.4 | 0.687 |
| CHX | -13.3 | 0.186 |

^1^ Tobit regression model-based latent means. ^2^ Wald contrasts computed from the Tobit regression model.

**Supplementary figure 1.** Generation of mouthwashes.

| **Preparation of EOS and CHX mouthwash** |
| --- |
| Sterile saline (B. Braun, Melsungen, Germany) was used to prepare EOS using an EOS generating device (Zarsten, Guandong, China) according to the manufacturer’s instructions. The free chlorine concentration was checked using spectrophotometric analysis (Hanna Instruments, Limena, Italy). Free chlorine concentrations were adjusted to 200 ppm by dilution with Milli-Q® water, and the pH was adjusted to 7.0 (corresponding to an HOCl:OCl- ratio of 75:25) using HCl. Independently generated batches were prepared before each rinsing stage. 0.12% CHX was prepared using a magistral pharmaceutical formulation. All mouthwashes were generated one day before administration and packaged into dark glass bottles with lids in volumes of 150 ml. |

**Supplementary figure 2**: CONSORT flow diagram.

***Arm 3***

***Arm 2***

***Arm 1***

***Allocation***

Randomized (*n* = 16)

Assessed for eligibility (*n* = 21)

Excluded (*n* = 5)

- Did not met inclusion criteria (*n* = 5)
- Declined to participate (*n* = 0)
- Other reasons (*n* = 0)
- Analyzed (*n* = 15)
- Excluded from analysis (did not complete all study arms) (*n* = 0)

***Analysis***

- Lost to follow-up (*n* = 0)
- Discontinued intervention (*n* = 0)

***Washout period***

Allocated to placebo (*n* = 5)

- Received allocated intervention (n = 5)
- Did not receive allocated intervention (*n* = 0)

Allocated to CHX (*n* = 6)

- Received allocated intervention (n = 5)
- Did not receive allocated intervention (drop out - personal reasons) (*n* = 1)

Allocated to EOS (*n* = 5)

- Received allocated intervention (n = 5)
- Did not receive allocated intervention (n = 0)

***Enrolment***

***Washout period***

Allocated to placebo (*n* = 5)

- Received allocated intervention (n = 5)
- Did not receive allocated intervention (*n* = 0)

Allocated to CHX (*n* = 5)

- Received allocated intervention (n = 5)
- Did not receive allocated intervention (*n* = 0)

Allocated to EOS (*n* = 5)

- Received allocated intervention (n = 5)
- Did not receive allocated intervention (n = 0)

***Follow-up***

Allocated to placebo (*n* = 5)

- Received allocated intervention (n = 5)
- Did not receive allocated intervention (*n* = 0)

Allocated to CHX (*n* = 5)

- Received allocated intervention (n = 5)
- Did not receive allocated intervention (*n* = 0)

Allocated to EOS (*n* = 5)

- Received allocated intervention (n = 5)
- Did not receive allocated intervention (n = 0)

**Supplementary figure 3.** Organoleptic properties and adverse effects.

| **Organoleptic properties and adverse effects** |
| --- |

The most distinct organoleptic properties were reported for CHX: 13 subjects described the mouthwash as bitter and 6 as unpleasant. Three subjects experienced a loss of taste lasting 1–3 hours after using CHX and one experienced a burning sensation in the oral cavity during rinsing. One described the taste as astringent and one as bleach. One subject noticed stained teeth at the gingival margin after 3 days of CHX use. Three subjects reported that their teeth felt clean even after 4 days of refraining from mechanical oral hygiene practices.

The taste of EOS was described as pool water or chlorine water by 7, as salty by 4, as bitter by 2, as unpleasant by 2 and as neutral by 2. Two subjects noticed that the mouthwash smelled of chlorine water. In addition, 4 subjects experienced a burning sensation in the oral cavity during rinsing. Four participants noticed ample dental plaque deposits on their teeth on day 2 of rinsing, and 1 on day 3.

The placebo mouthwash was described as having no or neutral taste by 11 subjects, while 2 found it bitter. Four participants noticed ample dental plaque deposits on their teeth on day 1 of rinsing, and two on day 2. One subject developed an aphthous lesion at the mucogingival junction below tooth no. 33 three days after commencing the placebo study arm; the lesion was monitored clinically on day 4 of the trial and resolved spontaneously within the next 5 days without the need for specific treatment.

**Supplementary figure 4.** Digital volumetric plaque analysis

| **VPI and AVPI** |
| --- |
| Intraoral scanning at the beginning (T0) and end (T4) of each study arm was used to obtain 3D models for digital volumetric dental plaque analysis. The process consisted of six steps: digital model acquisition; superimposition of digital models; computer aided determination of tooth surface margins; superimposition of tooth surfaces; visualisation and volumetric evaluation of dental plaque (Povšič et al, 2025). Post-processing was performed using 3D data processing software (GOM Inspect Suite 2022, GOM GmbH, Braunschweig, Germany). The volumetric plaque index (VPI; i.e. plaque volume in mm3) and adjusted volumetric plaque index (AVPI; i.e. plaque volume per surface area in mm3/mm^2^) were used to assess plaque volume.  *Povšič K, Munjaković H, Erčulj V, Fidler A, Gašperšič R. 3D Method for the Volumetric Evaluation and Visualisation of Dental Biofilms: A Proof-of-Principle Study. J Clin Periodontol. 2025 Sep 7. doi: 10.1111/jcpe.70019. Epub ahead of print.* |

**Supplementary figure 5.** Reaction mixtures for microbiological analysis.

| **Reaction mixture processing** |
| --- |
| The reactions mixtures consisted of 12.5 µL of Takyon Rox probe master mix dTTP blue (Eurogentec, Seraing, Belgium), 1 µL of each primer (IDT, Haasrode, Belgium) and probe (all DD probes, 5'-FAM [6-carboxyfluorescein] and 3'-TAMRA [6-carboxytetramethylrhodamine]; Eurogentec, Seraing, Belgium) and 4.5 µL of Milli-Q water. Cycle conditions were as follows: an initial step at 50°C for 2 min and 95°C for 10 min, followed by 45 cycles of 95°C for 15 s and 60°C for 1 min. |

**Supplementary figure 6.** Measurement of inflammatory mediator aMMP-8

| **aMMP-8 analysis** |
| --- |
| 1. Gingival crevicular fluid (GCF) samples were collected from the buccal surfaces of four Ramfjord teeth (16, 21, 36, and 41) using paper strips (Periopaper®, ProFlow, Amityville, NY, USA). The sampling site was prepared by the removal of excess saliva by air drying and relative isolation with cotton rolls. Next, each sterile strip was gently inserted into the gingival sulcus using tweezers until slight resistance was felt and left in place for 30 seconds, following previously described protocols. Any strips contaminated with blood or saliva were discarded (Hernandez et al, 2020). 2. Sample analysis was performed step by step in accordance with the manufacturer’s instructions for the PerioSafe® lateral-flow immunoassay kits (Dentognostics GmbH, Jena, Germany). For each participant, all paper strips were placed in a vial containing the elution buffer supplied with the kit. The vial was gently inverted five times to ensure complete immersion of the strips in the fluid. Subsequently, a dipstick was inserted into the elution fluid with the absorption zone facing downward until the liquid became visible in the readout window. The dipstick was then removed and positioned on the reader platform of the Ozalyser® device (Dentognostics GmbH, Jena, Germany), and the results were recorded from the display screen. aMMP-8 levels of less than 20 ng/ml were, as defined by the manufacturer, considered negative with regards to inflammation of the periodontal tissues; aMMP-8 levels of more than 20 ng/ml were considered as positive (Gupta et al, 2022).   *Hernández M, Baeza M, Contreras J, Sorsa T, Tervahartiala T, Valdés M, Chaparro A, Hernández-Ríos P. MMP-8, TRAP-5, and OPG Levels in GCF Diagnostic Potential to Discriminate between Healthy Patients', Mild and Severe Periodontitis Sites. Biomolecules. 2020 Oct 30;10(11):1500. doi: 10.3390/biom10111500.*  *Gupta S, Mohindra R, Singla M, Khera S, Kumar A, Rathnayake N, Sorsa T, Pfützner A, Räisänen IT, Soni RK, Kanta P, Jain A, Gauba K, Goyal K, Singh MP, Ghosh A, Kajal K, Mahajan V, Suri V, Bhalla A. Validation of a noninvasive aMMP-8 point-of-care diagnostic methodology in COVID-19 patients with periodontal disease. Clin Exp Dent Res. 2022 Aug;8(4):988-1001. doi: 10.1002/cre2.589.* |
